# The Emerging Complexity of γδT17 Cells

**DOI:** 10.3389/fimmu.2018.00796

**Published:** 2018-04-20

**Authors:** Duncan R. McKenzie, Iain Comerford, Bruno Silva-Santos, Shaun R. McColl

**Affiliations:** ^1^Department of Molecular & Cellular Biology, University of Adelaide, Adelaide, SA, Australia; ^2^Faculdade de Medicina, Instituto de Medicina Molecular, Universidade de Lisboa, Lisboa, Portugal

**Keywords:** γδ T cells, IL-17, T cell receptor signaling, migration, plasticity, immunological memory, translation

## Abstract

Preprogrammed IL-17-producing γδ T cells constitute a poorly understood class of lymphocytes that express rearranged antigen receptors but appear to make little use of them. γδT17 cells were first characterized as tissue-resident sentinels with innate effector function. However, ongoing research continues to reveal unexpected complexity to this unusual subset, including phenotypic plasticity, memory-like activity and unique migratory behavior. Despite these advances, at the core of γδT17 cell biology remain fundamental gaps in knowledge: Are γδT17 cells truly innate or has the importance of the T cell receptor been overlooked? How unique are they among IL-17-producing lymphocytes? How similar are these cells between mice and humans? We speculate that answering these unresolved questions is key to successful manipulation of γδ T cells in clinical settings.

## Introduction

Whereas conventional αβ T cells expressing diverse T cell receptors (TCRs) continuously patrol lymphoid tissues and extensively proliferate and differentiate to generate pathogen-tailored effector responses upon detection of cognate antigen, numerous innate-like lymphocyte subsets constitutively occupy barrier tissues and respond far more rapidly to tissue stress and infection. γδ T cells that produce interleukin 17 (IL-17, termed γδT17 or alternatively γδ17, Tγδ17) are one such population attracting increasing attention. Peripheral tissue localization coupled with preprogrammed effector function and a capacity for rapid antigen-independent activation enables γδT17 cells to respond within hours of infection. As such, γδ T cell-derived IL-17 is critical for control of pathogen load during the earliest stages of infection in a range of models. However, this innate-like response is not unique to γδT17 cells, as innate lymphoid cells (ILCs) and some invariant αβ T cell subsets also contribute to early production of Type 3 cytokines, which include IL-17, IL-22 and granulocyte-macrophage colony stimulating factor (GM-CSF). Thus, why these different lymphocyte subsets have co-evolved to fill the same protective niche remains unclear, although some of the features of γδT17 cells discussed throughout this review may highlight functions unique to these cells. Moreover, while γδT17 cells have been identified in humans, they exhibit some apparently fundamental differences from their murine counterparts that require further clarification before findings in mice may be exploited to understand human biology and ultimately influence clinical practice.

**Figure 1:**
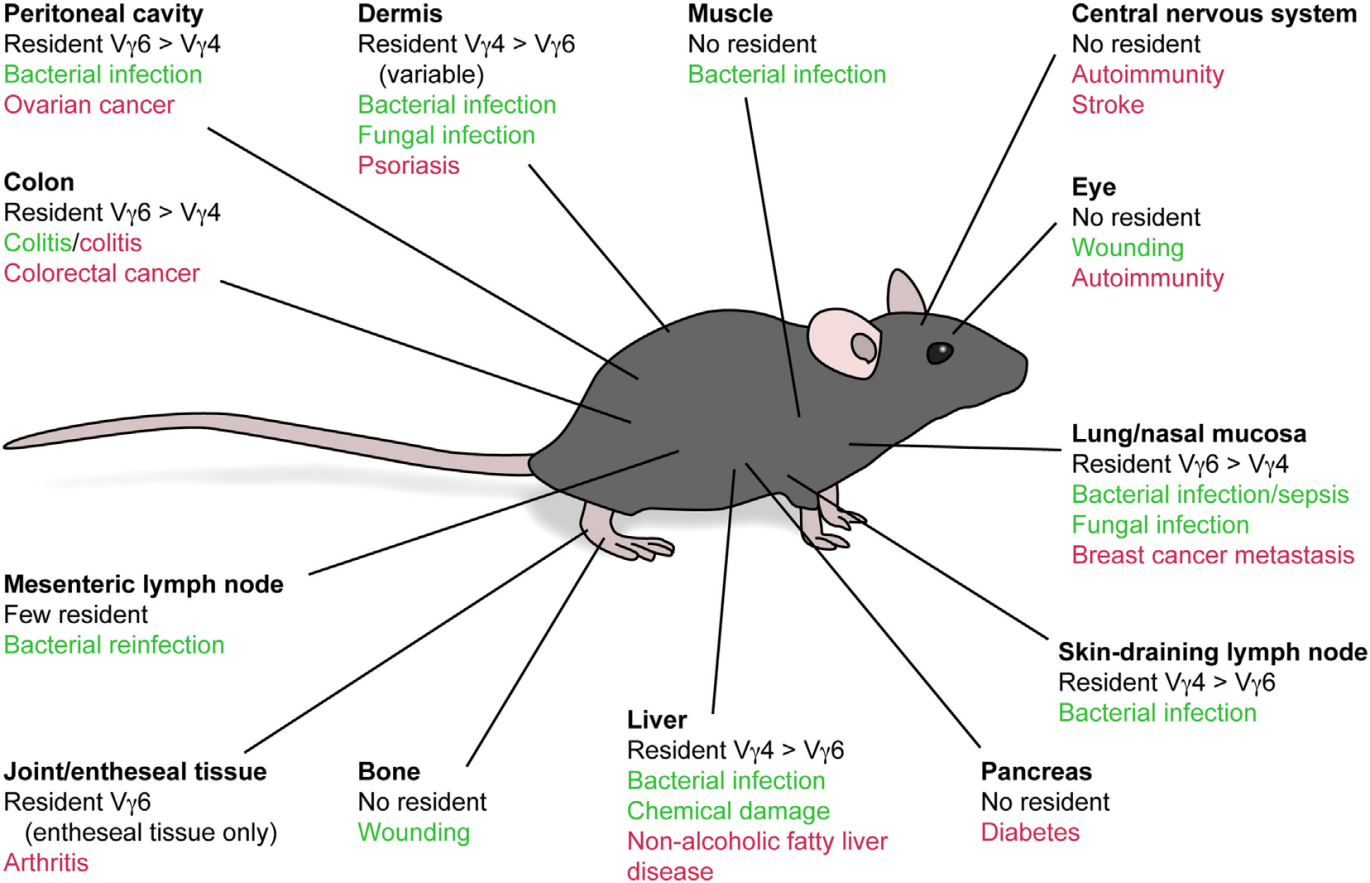
Beneficial and detrimental functions of local and infiltrating γδT17 cells. Vγ4^+^ and Vγ6^+^ γδT17 cells distribute to numerous peripheral tissues following development, although with differential bias. These cells are implicated in beneficial (green) and detrimental (red) immune responses both in these tissues and those that do not evidently harbor a resident γδT17 cell population. This suggests that migratory behavior of γδT17 cells, particularly during autoimmune conditions, exerts a strong influence on the outcome of inflammation.

γδT17 cells express receptors for the innate-derived inflammatory cytokines IL-23 and IL-1β, enabling immediate activation *in situ* following detection of invading microbes by myeloid and stromal cells (1–3). The contribution of γδT17 cells to antimicrobial immunity is most predominant in tissues harboring high frequencies of these cells at homeostasis: lung, skin, liver, peritoneal cavity, and lymph nodes (LNs) (Figure 1). However, aberrant γδT17 cell activity promotes autoimmune inflammation in numerous murine models (4). Unlike protective scenarios, many of these pathological responses involve target tissues that lack substantial local γδT17 cell populations, suggesting that γδT17 cells expand and subsequently home into autoimmune inflammatory foci. A key exception is psoriatic dermatitis, which manifests in the γδT17 cell-replete dermis. However, skin-resident γδT17 cells still appear to migrate between layers of the skin in this setting, and recent studies suggest a poorly understood interplay between local and infiltrating cells in the pathogenesis of skin inflammation (5, 6). γδT17 cell activity also promotes tumor growth in multiple murine models, which may arise from recruitment of myeloid cells and promotion of angiogenesis (7). The role of γδT17 cells in beneficial or detrimental immune responses has been extensively reviewed and will not be discussed further except where directly relevant (8).

γδT17 cells are further divided into two subsets as defined by the variable γ chain usage of their TCR. Those expressing the invariant Vγ6Vδ1 TCR strictly develop during embryogenesis and subsequently home to the dermis, lung, intestine, peritoneal cavity, and uterus (9). Alternatively, γδT17 cells expressing Vγ4 TCRs may develop in the adult thymus, are not invariant (although are fairly restricted) and represent only a fraction of the total Vγ4^+^ γδ T cell pool (10, 11). Vγ4^+^ γδT17 cells home to LNs, lung, liver, and the dermis alongside Vγ6^+^ cells, although the ratio of these two subsets in the dermal γδT17 cell population is variable and may be microbiota dependent (10, 12, 13). The contribution of particular γδT17 cell subsets to defense against infection or pathogenic activity during cancer often reflects the local subset bias at the effector site. Why two populations with such similar effector function develop separately and inhabit different tissues remains an open question. It is possible that the more tissue-biased Vγ6^+^ subset prioritizes immunosurveillance of barrier sites, while the lymphoid organ-skewed Vγ4^+^ subset serves as a pool that is mobilized to distal sites during local and systemic challenges, although this remains to be formally demonstrated. Intriguingly, these two populations can respond to distinct stimuli even within the same location, as demonstrated by dermal Vγ4^+^ and Vγ6^+^ cells which selectively expand following skin colonization with *Corynebacterium accolens* and *Staphylococcus epidermidis*, respectively (14).

Understanding of γδT17 cell development and function is far from complete, as we know little about many key aspects of their basic biology that are well established in conventional T cells. For example, the function and specificity of the Vγ4 and Vγ6 TCRs remain undefined. It is still unknown whether ligand–TCR interactions are relevant to thymic selection or peripheral function of γδT17 cells, nor whether potential ligands are host-derived or foreign. Understanding how and when the TCR functions in γδT17 cell biology should clarify whether these cells occupy a niche closer to ILCs or invariant αβ T cells in terms of fundamental biology and may shed light on the recent descriptions of memory-like γδT17 cell responses during infection and chronic inflammation (6, 15). Moreover, elucidation of the previously unappreciated plasticity and migratory dynamics of γδT17 cells is underway but remains incompletely defined (16, 17). Here, we review the current state of knowledge in these emerging concepts, in the form of the key questions that should be answered to progress knowledge of γδT17 cells toward clinical application.

## What is the Role of TCR Signaling in γδT17 Cell Development?

While recent work has somewhat clarified the role of TCR signaling in γδT17 ontogeny, whether true ligand-driven selection, akin to that experienced by αβ T cells, occurs during their thymic development remains unclear. A number of transcription factors, cytokine signals, and surface receptor interactions are essential for γδT17 cell development and have been reviewed recently elsewhere (18). However, it is worth reiterating that γδT17 cell ontogeny requires RORγt and TGF-β, two factors also crucial to *de novo* polarization of Th17 cells from naïve αβ T cells, suggesting that the induction of the Type 3 program in these cell types is fundamentally conserved despite occurring under different conditions, in different sites and with some divergent signal requirements (19, 20).

### Shifting Views on Instructive TCR Signaling in γδT17 Cell Development

Early studies suggested that γδT17 cells do not receive antigen-driven TCR signals development, as TCR engagement promotes alternate fates. Initially, the Chien laboratory proposed that TCR activation in the thymus drives γδ T cells toward the interferon (IFN)-γ program (γδT1) at the expense of the γδT17 pathway (21). This conclusion derived from the observation that unlike γδT1 cells, peripheral γδT17 cells lack surface CD122 expression, a marker previously associated with antigen recognition by αβ thymocytes (22). Further support for this concept arose from studies of dendritic epidermal T cells (DETCs). Mice with a loss-of-function mutation in *Skint1*, a butyrophilin-like transmembrane protein, lack prototypic Vγ5Vδ1^+^ DETCs as their precursors fail to mature in the embryonic thymus (23). However, the immature DETC precursors in these mice exhibit an abnormal γδT17 phenotype rather than the wild-type IFN-γ/IL-13 program (24). This may suggest that the IL-17 fate is the default program of γδ T cells, which is normally avoided by instructive signals such as Skint1. However, as it remains unknown whether Skint1 is a Vγ5Vδ1 TCR ligand, this does not demonstrate that TCR signaling *per se* instructs developing γδ T cells away from the IL-17 fate, nor whether this concept applies to naturally developing γδT17 cells.

More recently, the concept that γδT17 cells do not experience TCR engagement in the thymus has been challenged by three key studies of mice with genetic deficiencies in this pathway. First, there is a striking lack of Vγ4^+^ and Vγ6^+^ γδT17 cells in mice with reduced TCR signal strength due to a hypomorphic mutation in the TCR signaling intermediate Zap70 (25). Second, mice haploinsufficient for TCR signaling components CD3γ and CD3δ have reduced numbers of Vγ6^+^ but not Vγ4^+^ γδT17 cells (26). Third, mice deficient in Syk, a kinase classically associated with B cell receptor signaling, and downstream PI3 kinase, lack all γδT17 cells. Strangely, Zap70-deficient mice here showed a deficit only in Vγ6^+^ γδT17 cells, whereas both Vγ4^+^ and Vγ6^+^ cells were affected in the hypomorphic mutant (27). A solid explanation for the differential effects of these mutations upon Vγ4^+^ γδT17 cells is lacking. However, it has been posited that developing αβ T cells undergo stronger TCR signaling during the fetal period, which may suggest that fetal-derived Vγ6^+^ cells require higher threshold signaling than their adult Vγ4^+^ counterparts (28). Alterations in the Vγ6^+^ to Vγ4^+^ γδT17 cell ratio are also observed in mice with mutations affecting cortical thymic epithelial cell function (29, 30).

Further dissection of the specific nature of TCR signaling during γδT17 cell development has stemmed from more detailed understanding of surface marker expression during this process. Coffey and colleagues identified surface CD73 as a selective marker of TCR–ligand experienced γδ T cells by interrogating KN6 γδ-TCR transgenic thymocytes, which recognize known ligands T10 and T22 (31). As the majority of γδT17 cells in the wild-type adult thymus are CD73^+^, this suggested that TCR–ligand interaction naturally occurs during their ontogeny. Furthermore, this study showed that KN6 transgenic γδT17 cells do not develop in the absence of T10 and T22 (31). Subsequently, fetal γδ thymocytes lacking CD24, CD44, and CD45RB expression were identified as the common precursor of both CD44^+^ γδT17-committed cells and CD45RB^+^ γδT1-committed cells. However, antibody-mediated TCR crosslinking drives these precursors selectively to the IFN-γ program, inhibiting γδT17 cell development (32). This report may therefore explain the apparently contradictory results from earlier studies by clarifying that γδT17 cells receive “weaker” TCR signals than other subsets. Together, these studies provide clear evidence that TCR engagement of a certain nature is required for γδT17 cell development. It is likely that the discrete signaling pathways engaged by different modes of TCR activation are crucial for the successful programming of γδT17 cell effector function, although this requires further investigation.

### TCR-Independent Facets of γδT17 Cell Development

Further dissection of γδT17 cell development has revealed that a requirement for TCR signaling may only exist for certain elements of this process. While most developing γδT17 cells express the antigen-experience marker CD73 in adulthood (31), this is not the case during early life. Anderson and colleagues recently determined that the majority of both Vγ4^+^ and Vγ6^+^ γδT17 cells developing during the fetal and perinatal period progress directly from a CD24^+^ immature to CD24^−^ mature phenotype without ever inducing CD73 (33). These CD73^−^ γδT17 cells are completely dependent upon the transcription factor HEB for induction of γδT17 cell lineage-specifying factors *Sox4, Sox13*, and *Rorc*. Mature CD73^−^ γδT17 cells are also detectable in peripheral tissue, although the majority of tissue γδT17 cells remain CD73^+^ (33). Although direct analysis of TCR signaling was not undertaken, this report suggests that while most postnatally derived Vγ4^+^ γδT17 cells experience thymic antigen, γδT17 cells developing during the fetal period do not. It will be important to reconcile conclusions from CD73 studies with mice deficient in TCR signaling intermediates to clarify whether particular subpopulations of γδT17 cells show distinct requirements for TCR signals during development.

Taken together, the evidence outlined so far suggests that the emergence of mature γδT17 cells from the thymus is largely TCR dependent. An important but distinct question is whether induction of IL-17 effector function in normally developing γδ T cells is explicitly dependent upon TCR engagement. IL-17 expression in the fetal thymus coincides with *Tcrd* locus opening and rearrangement, before the expression of a functional TCR in T cell-committed progenitors (34). Moreover, the expression of key γδT17 lineage-specifying transcription factors in developing Vγ4^+^ cells is largely unaffected by deficiency of ITK, a protein crucial for γδ-TCR signal transduction (35). These reports thus far indicate that the IL-17 effector program arises before, and therefore independently of, expression of the γδ-TCR. In context of the prior discussion, this suggests that any role for TCR signaling in γδT17 cell development is subsequent to the IL-17 fate decision, instead promoting ensuing survival, proliferation, and/or further maturation.

Importantly, the most mechanistic studies to date identify a role for TCR signaling but not necessarily ligand encounter during γδT17 cell thymic development. As solid information regarding the ligand(s) of the Vγ4 and Vγ6 γδT17 TCRs is lacking, it is difficult to distinguish TCR signals driven by ligation of physiological antigen from TCR assembly driven signals, which have been reported (36). Therefore, the identification of γδ-TCR ligands remains critical for understanding thymic selection, preprogramming, and antigen specificity in the context of peripheral responses.

## Do Mature γδT17 Cells Use Their TCR?

Whether TCR signaling fulfils an important physiological function in mature murine γδT17 cells is unclear, a critical question to answer given the more obvious function of human γδ-TCRs. Murine γδT17 cells can be activated solely by innate-derived cytokines, predominantly IL-23 and IL-1β, but also IL-7 and IL-18 (2, 37, 38). This is reminiscent of Th17 cells, which can also be activated independently of TCR stimulation by IL-23 and IL-1β once polarized (39). This observation is consistent with the programmed “effector memory”-like phenotype of γδT17 cells. However, as TCR signaling is patently implicated in Th17 effector function, it also hints that the TCR may modulate γδT17 cell activity when combined with innate signals.

### Evidence for TCR Signaling in Preprogrammed γδT17 Cell Responses

While not essential, it is clear that crosslinking of the TCR by anti-CD3 or pan anti-γδ-TCR antibodies does activate γδT17 cells. *In vitro* TCR stimulation alone is sufficient to induce IL-17 secretion by γδ T cells, and TCR signals enhance the amount of IL-17 produced in response to innate cytokines (21, 40–42). In addition, TCR crosslinking enhances IL-7-driven proliferation of γδT17 cells and promotes their efficient *in vitro* expansion (17, 38). *In vivo*, administration of anti-Vγ4 antibodies exacerbates experimental autoimmune encephalomyelitis (EAE) symptoms as it activates pathogenic Vγ4^+^ γδT17 cells rather than depleting them (43). However, while suggestive, these data do not prove a physiological function for TCR signaling in γδT17 cell responses. Several studies (discussed below) have utilized the Nur77-GFP reporter mouse, commonly used to measure αβ-TCR signal strength, to determine whether TCR signaling underpins γδT17 memory-like responses. However, *in vitro* stimulation with IL-23 and IL-1β alone also induces some level of reporter expression (6), and so additional methods are required to investigate whether physiological Vγ4^+^ or Vγ6^+^ TCR signaling occurs during γδT17 cell responses *in vivo*. Inducible deletion of the γδ-TCR in mature, fluorescently labeled γδ T cells would help to address this important question.

A key clarification is that the threshold required for activation of downstream TCR signaling is significantly greater in γδT17 cells than other lymphoid γδ T cell subsets. CD27^+^ γδ T cells, which are biased toward IFN-γ production and are predominantly found in lymphoid organs, undergo a conventional αβ T cell-like response to TCR crosslinking, showing rapid Ca^2+^ flux and phosphorylation of Erk. By contrast, very little response to this stimulation is observed in γδT17 cells, and a substantially higher concentration of crosslinking antibody is required to induce Nur77-GFP expression (25). A similar hyporesponsive TCR is documented for DETCs and a subset of innate-like γδT1 cells. Considering that tonic TCR engagement is observed in DETCs (44), it is possible that a higher signaling threshold is needed to ensure that they are only activated upon upregulation or relocalization of cognate self-antigen during tissue stress. This in itself is merely speculative, so whether the higher TCR threshold in γδT17 cells reflects the nature of their putative antigen(s) is unknown.

A recent study reported that γδT17 cells appear to directly recognize microbiota-derived lipids presented by the non-classical MHC molecule CD1d (45). The maintenance of peritoneal cavity and gut-associated γδT17 cells is dependent upon the microbiome, as they are diminished in mice treated with antibiotics or raised in germ-free conditions (46). Tian and colleagues extended these findings to hepatic γδT17 cells, which are similarly depleted upon antibiotic treatment (45). Moreover, hepatic γδT17 cells are deficient in *Cd1d^−/−^* mice, independent of microbiota composition. CD1d is well known to present microbial-derived lipids to NKT cells expressing an invariant αβ-TCR, although it has also been crystallized presenting lipid to a human Vδ1^+^ TCR (47, 48). Murine hepatic, but not splenic, γδT17 cells bind CD1d tetramers loaded with various bacterial lipids, and when purified are activated *in vitro* by hepatocytes in a CD1d-dependent manner (45). While no biochemical data have yet been reported to confirm presentation of lipids directly to murine γδT17 TCRs, it will be of great importance to pursue this intriguing possibility as it is not only a strong lead in the hunt for γδ-TCR ligands but may be immediately relevant to human γδ T cells.

### Induction of γδT17 Effector Function in Peripheral γδ T Cells

While the general consensus is that γδT17 cell function is preprogrammed, some notable studies have documented inducible γδT17 cells that develop from naïve precursors following antigen engagement. These instances are intriguing because they more closely reflect the human system, where γδT17 cell effector phenotype can be induced from “naïve” precursors upon TCR stimulation and exposure to appropriate cytokines (49, 50). Chien and colleagues reported populations of murine γδ T cells specific for phycoerythrin (PE) and haptens cyanine 3 and 4-hydroxy-3-nitrophenylacetyl, which induce key γδT17 genes including *Il17a, Il17f, Rorc*, and *Ccr6* following antigen-specific immunization (41, 51). Moreover, immunization with PE drives upregulation of *Il23r* and *Il1r1* in PE-specific γδ T cells, suggesting that IL-23 and IL-1β may boost antigen-driven IL-17 production in these cells. These studies were also notable in that they identified the first genuine γδT17 TCR ligands, unequivocally demonstrated by surface plasmon resonance. However, the natural frequency of PE- and hapten-specific γδ T cells is on the order of 0.1% of splenic γδ T cells, which more closely reflects clonal frequencies of naïve conventional antigen-specific αβ T cells than the highly restricted TCR diversity observed in “natural” γδT17 cells. How these rare “inducible” γδT17 cell clones, which express diverse Vγ1 and Vγ4 TCRs, relate to the 100-fold more abundant invariant and semi-invariant Vγ6^+^ and Vγ4^+^ γδT17 cells is unclear.

Conversely, two recent complementary reports identified inducible γδT17 cells on a larger scale. Both used radiation bone marrow chimeras to reveal *de novo* differentiation of γδT17 cells from precursors in the periphery, as thymus-derived “natural” γδT17 cells do not arise from adult bone marrow progenitors in many laboratories. First, induced γδT17 cells were identified during EAE following bone marrow reconstitution of *Tcrd^−/−^* hosts (42). These were dependent upon IL-23 signaling alone, which is somewhat unexpected given the requirement of naïve αβ T cells to first experience IL-6 to upregulate the IL-23 receptor during Th17 polarization (52). Second, IL23R^+^ γδT17 cells developed from peripheral IL23R^−^ γδ T cells during imiquimod (IMQ)-induced psoriasis (53). In this case, both IL-23 and IL-1β signals were essential. Notably, the former report determined that while TCR stimulation was not essential for induction of γδT17 cells *in vitro*, it did synergize with cytokine signals to promote their development. The latter report utilized TCR stimulation throughout, thus it is unclear whether it is essential in that case. It will be important to determine the broader contribution of inducible γδT17 cells to murine pathophysiology, given their more immediate relevance to humans as discussed below.

## Are γδT17 Cells Capable of Memory Responses?

Conventional memory responses involve the persistence of a quiescent population of antigen-specific effector T or B cells following resolution of infection, which rapidly expand during antigenic rechallenge and efficiently control reinfection. Therefore, a central tenet of classical memory is antigen specificity. However, as discussed earlier, γδT17 cell antigens are unknown and may even be irrelevant to their biology. Thus, it is fascinating that reports continue to emerge of enhanced γδT17 cell frequency and activity upon secondary rechallenge in bacterial infection. In addition, memory-like γδT17 cell responses are observed during psoriatic dermatitis models, where there is no immunizing antigen (although stress-induced self-antigens would be abundant).

A “memory” response involving γδT17 cells was first documented in the mesenteric LNs of mice previously infected with oral *Listeria monocytogenes*. Here, Vγ6^+^ cells remained at elevated frequencies following primary infection and proliferated rapidly when specifically rechallenged with the same pathogen only *via* the same route (15). This “memory” response was later shown to be dependent on IL-17-driven formation of γδT17 and myeloid cell clusters around *L. monocytogenes* replication foci (54). As purported antibody-mediated internalization of the γδ-TCR inhibited this recall Vγ6^+^ γδT17 cell response *in vivo*, it was suggested that memory-like γδT17 cells are reactivated in a TCR-dependent manner (15). However, more definitive demonstration of a TCR-specific response is lacking in this scenario.

This memory-like behavior has subsequently been observed in other bacterial infections. First, Vγ6^+^ γδT17-dependent “memory” responses against *Staphylococcus aureus* rechallenge were identified in the peritoneal cavity, and transfer of peritoneal γδ T cells from previously challenged mice led to reduced bacterial load in newly challenged recipients. Here, activation of Vγ6^+^ “memory” cells *in vitro* by coculture with infected macrophages is not inhibited by blockade of IL-23 or IL-1β signaling, indirectly suggesting that the TCR may be involved (55). Most recently, expanded lung Vγ4^+^ γδT17 cells were shown to proliferate more rapidly upon rechallenge with *Bordetella pertussis*, and these memory-like cells, when purified, respond to heat-killed *B. pertussis in vitro* (56). These examples demonstrate that both Vγ4^+^ and Vγ6^+^ γδT17 cells can remain in target tissues at higher frequency following resolution of infection, and therefore expand more rapidly to control pathogen colonization upon rechallenge. However, whether this represents *bona fide* TCR-dependent, antigen-specific memory or instead to corresponds to memory-like behavior observed in natural killer (NK) or myeloid cells remains to be established (57, 58).

γδT17 cell memory-like responses have also been observed during IMQ-induced psoriasis, where activated Vγ4Vδ4^+^ cells redistribute to distal uninflamed skin, thus driving enhanced pathology upon subsequent challenge of previously unaffected skin (5, 6). Both studies reporting this phenomenon demonstrated induction of Nur77, a marker of early TCR signaling, specifically within the “memory” population upon rechallenge. However, Nur77 is also induced by IL-23 and IL-1β signaling alone *in vitro*, suggesting that these results should be cautiously interpreted. Regardless, the selective response of γδT17 cells bearing a specific γδ-TCR chain pairing, given that Vγ4 may pair with multiple δ chains, does hint at a TCR-selective response. Memory-like skin Vγ4^+^ γδT17 cells also show elevated IL-1R1 expression, suggesting that in this scenario, heightened sensitivity to cytokine stimulation may contribute to the recall behavior (6). This experimental system also uncovered novel γδT17 trafficking dynamics which will be discussed below.

From current evidence, it is clear that γδT17 cells can respond with heightened kinetics upon repeated inflammatory challenge or infection. These responses profoundly influence the outcome of inflammation, be it worsening psoriatic dermatitis or enhancing bacterial clearance. Determining whether these phenomena are examples of true immunological memory will require comprehensive demonstration of a TCR- and antigen-dependent response. If this is in fact the case, it will be an excellent opportunity to elucidate the antigens recognized by γδT17 TCRs. They are likely to be either self-stress signals and/or conserved bacterial products, given the broad reactivity of Vγ6^+^ cells bearing invariant receptors. Alternatively, these memory-like responses may more resemble trained immunity, the memory-like behavior observed in NK cells, myeloid cells, and most recently epithelial stem cells, due to epigenetic changes facilitating more powerful activation upon re-exposure to inflammatory stimuli (57–59). Further research into this area will be of great use to the field.

## When and How do γδT17 Cells Exhibit Plasticity?

While generally “rigid” in effector function, some reports of plasticity have emerged suggesting that γδT17 cell responses can be fine tuned over the course of inflammation. Their αβ counterparts, CD4^+^ Th17 cells, display marked phenotypic plasticity during *in vivo* responses. Although IFN-γ is the defining effector cytokine produced by Th1 cells, Th17 cells are induced to co-express IFN-γ and IL-17 by signals such as IL-12 and IL-23 (60, 61). Furthermore, by generating a mouse capable of permanently marking cells that had transcribed the *Il17a* locus at some point in their history, Stockinger and colleagues discovered that the majority of central nervous system-infiltrating, IFN-γ-producing CD4^+^ T cells during EAE were formerly Th17 cells that had subsequently extinguished IL-17 production (62). IFN-γ production by Th17 cells is dependent upon transcription factors T-bet, Runx1, and Runx3 (62, 63). Notably, Th17 cells do not lose IL-17 nor gain IFN-γ expression during cutaneous fungal infection, suggesting that a particular inflammatory milieu dictates the plasticity of Th17 cells. Analysis of γδ T cells alongside Th17 cells in EAE and fungal infection revealed negligible plasticity in either setting (62).

These findings cemented the view that γδT17 cells are fixed in phenotype until several studies began to describe IFN-γ^+^IL-17^+^ γδ T cells in select scenarios. First, Vγ6^+^ “memory” γδT17 cells in oral *L. monocytogenes* rechallenge were shown to co-produce IFN-γ, alongside induction of the classically Type 1-associated chemokine receptor CXCR3 (15, 54). Subsequently, a large proportion of late-stage tumor-infiltrating Vγ6^+^ γδT17 cells in a peritoneal model of ovarian cancer were also identified to produce IFN-γ (16). These reports of *in vivo* plasticity of γδT17 cells support *in vitro* evidence of IFN-γ production by γδT17 cells when stimulated with IL-23 and IL-1β (40). As both described examples hitherto feature Vγ6^+^ γδT17 cells, whether this subset is more plastic than Vγ4^+^ γδT17 cells is unclear. It is important to clarify at this point that while plasticity of γδT17 cells in the above scenarios is clear, γδT17 cells do not produce IFN-γ in the majority of settings investigated, suggesting that this behavior is tightly regulated.

Insight into the potential for γδT17 cells to induce a Type 1 phenotype arose from comparative genome-wide epigenetic analysis of CD27^+^ (γδT1-enriched) and CD27^−^ (γδT17-enriched) γδ T cells (16). As anticipated, γδT1 cells exhibit permissive H3K4 dimethylation marks upon characteristic genes *Ifng, Tbx21*, and *Eomes* and repressive H3K4 trimethylation on γδT17 lineage genes *Rorc, Il17a, Il17f*, and *Il22*. However, γδT17 cells display permissive marks not only on γδT17 lineage genes as expected but also on *Ifng* and *Tbx21*. These data indicate that γδT17 cells are epigenetically “primed” to induce γδT1 factors, but not *vice versa*. It will be insightful to elucidate the stimuli responsible for inducing T-bet expression and IFN-γ production in γδT17 cells, as this may influence their protective and/or pathogenic behavior akin to Th17 cells. While IL-23 and IL-1β stimulation promotes IFN-γ secretion by γδT17 cells in some reports, additional signals are likely required, as these two cytokines direct γδT17 cell activity during *in vivo* settings both with and without evidence of plasticity. Moreover, while IL-12 promotes IFN-γ expression by Th17 cells, γδT17 cells do not express its receptor and do not respond in this manner (61, 64). While the upstream signals are somewhat unclear, it is now evident that γδT17 cell plasticity is restricted by post-transcriptional mechanisms. Specifically, γδT17 cells were recently found to selectively express high levels of microRNA miR-146, which targets Nod1 to suppress IFN-γ production (65). Considerable co-expression of IL-17A and IFN-γ is evident from *in vitro* polarized human γδT17 cells (49, 50), therefore understanding the mechanism and relevance of γδT17 cell plasticity is another worthy pursuit in the path to therapeutic manipulation of human γδ T cells.

## How and Why do γδT17 Cells Establish Their Migration Patterns?

γδT17 cells may be considered innate-like cousins of tissue-resident memory T cells as they similarly inhabit barrier tissues in a poised state, primed to initiate inflammation upon microbial (re)invasion. However, even the earliest studies implied that γδT17 cells are distinct in their ability to traffic to distant sites. Indeed, several key murine autoimmune models in which γδT17 cells are implicated involve their migration to and infiltration of target sites that do not harbor resident populations (Figure 1). Moreover, new evidence suggests that γδT17 cells adopt an unusual hybrid homeostatic migration pattern that spans true tissue residency and free naïve αβ T cell recirculation.

γδT17 cells are selectively enriched in skin-draining lymph nodes (sLNs) but are also detected in circulation. Cyster and colleagues first hinted at constitutive γδT17 cell trafficking by detecting dermis-derived Vγ4^+^ T cells in sLNs in under homeostatic conditions, using Kaede photoconvertible reporter mice (66). Subsequently, sphingosine-1-phosphate antagonism demonstrated that the circulating γδT17 cell population is LN-derived (67). This loop is completed by recruitment of blood-borne γδT17 cells back into the dermis by constitutively expressed chemokine receptor CCR6, probably in concert with cutaneous lymphocyte antigen (CLA) (6, 10, 17). Whether CCR6 directs γδT17 cells to other uninflamed barrier tissues is unclear, although their frequency is unaltered in the lung and liver of *Ccr6^−/−^* mice (17). CCR6 also positions Vγ4^+^ cells in the LN subcapsular sinus and is critical for their response to lymph-borne *S. aureus* (68). However, recent parabiosis experiments have demonstrated that while γδT17 cells indeed move between sLNs, blood, and dermis, their trafficking is fairly restricted compared with αβ T cells (68, 69). This limited motility appears to be imposed by LN macrophages, whose blebs are acquired by γδT17 cells at steady state (70).

γδT17 cells constitutively express a range of homing receptors which enable their rapid recruitment to sites of inflammation (Table 1). In particular, CCR2, a receptor predominantly associated with mononuclear phagocyte migration, drives γδT17 cell infiltration of numerous inflamed tissues and is crucial for their protection against *S. pneumoniae* infection (6, 17, 71). Whereas most unambiguous descriptions of γδT17 cell trafficking during inflammation involve sites lacking a resident population, findings in *S. pneumoniae* infection and psoriasitic dermatitis models suggest that blood-borne γδT17 cells also infiltrate tissues already hosting local γδT17 cells. Intriguingly, dermal Vγ4^+^ γδT17 cells migrate from inflamed skin to draining LNs during IMQ psoriasis, proliferate, and then migrate both to the original inflamed tissue and to distal uninflamed skin (5, 6). As discussed earlier, this memory-like behavior appears to be based upon increases in tissue γδT17 cell frequency, indicating that migratory characteristics define the influence of γδT17 cells on the outcome of inflammation. While γδT17 cell redistribution to unaffected skin predisposes that area to more severe inflammation, the influence of LN-expanded γδT17 cell homing back to already inflamed skin is unclear, as retention of these cells in LNs by sphingosine-1-phosphate antagonism does not affect the progression of skin inflammation (72).

**Table 1:** Homing receptors involved in murine γδT17 cell migration.

Homing receptor*Ligands*	Tissue	Setting	Evidence	Reference
CCR2 *CCL2, CCL7, CCL12*	Skin	IMQ psoriasis	*Ccr2^−/−^* cell transfer	(6)
	Joints	*Il1rn^−/−^* arthritis	CCL2 neutralization	(71)
	CNS	EAE	*Ccr2^−/−^* cell transfer	(17)
	Tumor	B16 melanoma	*Ccr2^−/−^* cell transfer	(17)
		KEP breast cancer	CCL2 neutralization	(73)
	Nasal mucosa	*S. pneumoniae*	*Ccr2^−/−^* cell transfer	(17)

CCR6 *CCL20*	Skin	Homeostasis	*Ccr6^−/−^* cell transfer	(10)
			*Ccr6^−/−^* cell transfer	(17)
		IL-23 psoriasis	*Ccr6^−/−^* mice, CCL20 neutralization	(74)
		IMQ psoriasis	*Ccr6^−/−^* mice	(75)
			CCR6 antagonist, *Ccr6^−/−^* mice	(76)
	Cornea	Corneal abrasion	CCL20 neutralization	(77)
	Liver	CCl_4_, methionine–choline-deficient fibrosis	*Ccr6^−/−^* mice	(78)
	Brain	Stroke	*Ccr6^−/−^* mice	(79)

CCR9 *CCL25*	Lung	OVA challenge	CCL25 neutralization	(80)

CXCR3 *CXCL9, CXCL10, CXCL11*	mLN	*Listeria monocytogenes* rechallenge	CXCR3 neutralization	(54)

S1P_1_ *S1P*	Blood	Homeostasis	S1P_1_ antagonist	(67)
	Skin (*via* blood)	IMQ psoriasis	S1P_1_ antagonist	(6, 67)
	CNS (*via* blood)	EAE	S1P_1_ antagonist	(67)

α_4_β_7_ *MadCAM-1, VCAM-1*	Lung	OVA challenge	α_4_β_7_ neutralization	(80)

*IMQ, imiquimod; CNS, central nervous system; EAE, experimental autoimmune encephalomyelitis; IL-23, interleukin 23; OVA, ovalbumin; mLN, mesenteric lymph node*.

Unlike conventional T cell responses, which involve induction of inflammatory homing receptors during time-consuming expansion and polarization of effector cells, γδT17 cells constitutively express both homeostatic and inflammatory chemokine receptors. Unusually, they do not express the typical homeostatic receptor CCR7, and so most likely can only enter LNs from afferent lymph rather than directly from circulation (81). Instead γδT17 cells express CCR6, which is an unusual receptor as it directs recruitment of lymphocytes and myeloid cells both to homeostatic sites and inflamed tissues (82). It is important to clarify that the “homeostatic” sites where the sole CCR6 ligand CCL20 is expressed may not necessarily be uninflamed in the technical sense, as these mucocutaneous tissues are constantly exposed to environmental and microbial stress. Nevertheless, in multiple inflammatory scenarios, γδT17 cells downregulate CCR6 expression rapidly upon activation. This loss of CCR6 is beneficial for homing during inflammation as it prevents recruitment to uninflamed skin and thereby concentrates their homing toward inflamed tissues (17). However, CCR6 is implicated in the recruitment of γδT17 cells to inflammatory lesions in several scenarios, such as psoriasis, liver inflammation, and corneal damage, suggesting that modulation of its expression is context specific (76–78). Although CCR6 has been suggested to influence γδT17 cell migration during skin inflammation (Table 1), activated γδT17 cells either emigrating from inflamed dermis during psoriasis or migrating into inflamed epidermis during a transgenic model of oncogenesis have lost CCR6 expression (66, 83). It will be useful to reconcile these results given the expression of CCR6 by human skin-infiltrating γδ T cells, as discussed below. By contrast, memory-like Vγ6^+^ γδT17 cells in oral *L. monocytogenes* infection upregulate CXCR3 expression (54), which may be linked to their plasticity toward the IFN-γ program rather than an intrinsic property of chronically activated γδT17 cells.

**Figure 2:**
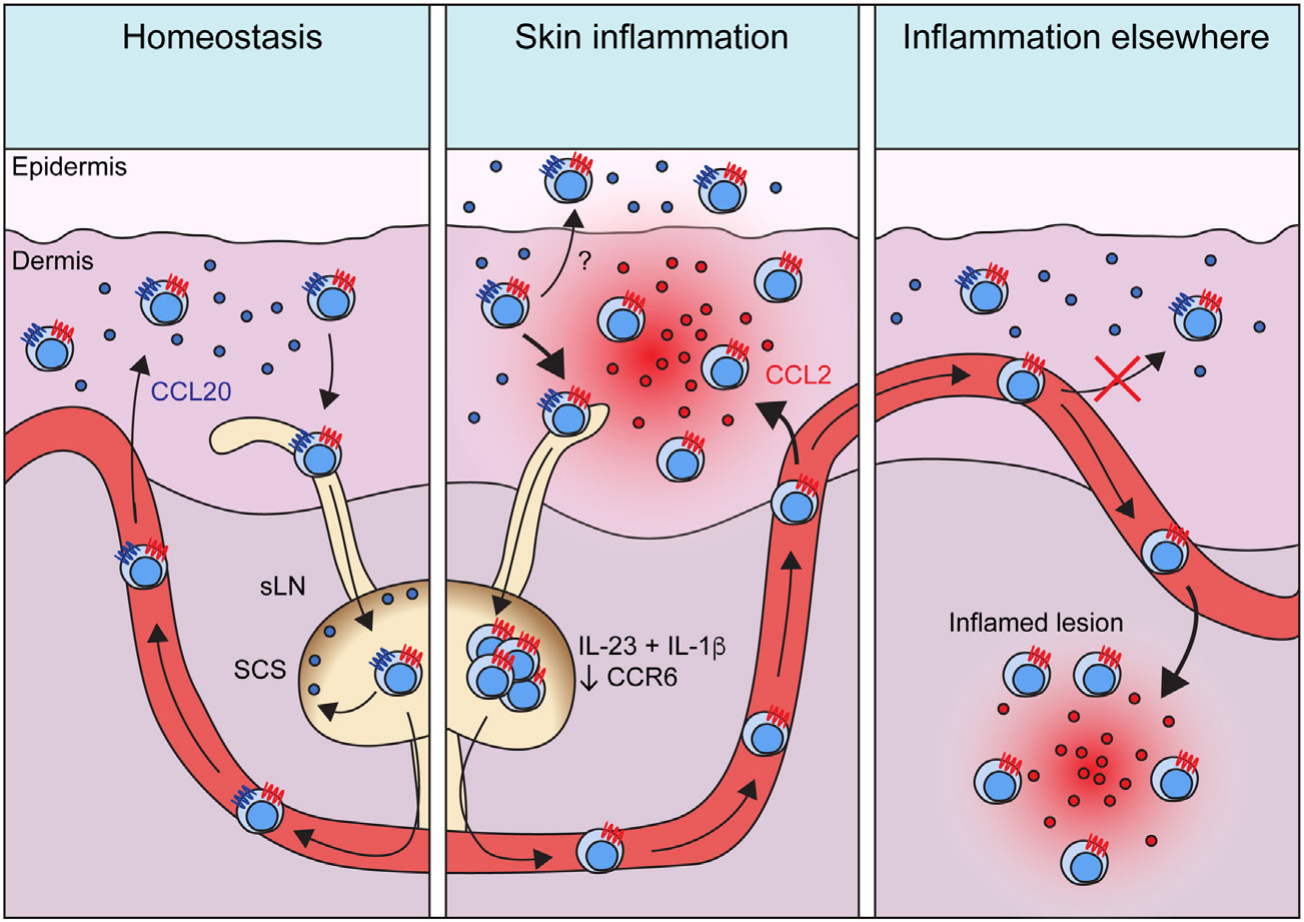
Migratory dynamics of γδT17 cells. Under homeostasis, γδT17 cells largely reside in barrier tissues such as the dermis, but also drain slowly into sLNs and are detectable in the blood. Circulating γδT17 cells return to the skin using CCR6 which directs them toward CCL20 expressed in the dermis. CCR6 also positions γδT17 cells in the sLN SCS to scan for invading microbes. During skin inflammation, γδT17 cell trafficking from the dermis to sLNs is increased. γδT17 cells undergo proliferation driven by IL-23 and IL-1β in sLNs, where they lose CCR6 expression. Activated and expanded γδT17 cells then home *via* the blood to inflamed skin using CCR2, which senses ligands such as CCL2 induced during inflammation. CCR6 probably recruits γδT17 cells into the epidermis during skin inflammation, but how its expression is maintained in this scenario is unknown. During inflammation in other peripheral organs, γδT17 cells similarly proliferate in LNs *via* IL-23 and IL-1β and become CCR6^−^. They then traffic *via* circulation to infiltrate the inflamed site *via* CCR2. Loss of CCR6 expression is required for optimal γδT17 cell recruitment to such inflammatory sites, as it prevents activated γδT17 cells from instead homing to unaffected dermis. Abbreviations: sLN, skin-draining lymph node; SCS, subcapsular sinus.

Despite advances in elucidating when and how γδT17 cells migrate, we still lack a solid understanding of why they establish such patterns. Dermal γδT17 cells are intrinsically motile, which may facilitate their surveillance of the skin (84). The purpose of draining to sLNs *via* afferent lymphatics, entering circulation, and returning to skin is less clear. It is not obvious that γδT17 cells need to scan LNs for antigen, especially considering their highly restricted TCRs. Instead, this process may serve to constantly redistribute γδT17 cells to other skin sites or maintain a constant peripheral blood pool that could act as an immediate reservoir of effector cells when inflammation arises. During tissue inflammation, γδT17 cells proliferate in draining LNs and home toward the inflammatory foci (85). While largely observed in autoimmune scenarios where the target tissue lacks resident γδT17 cells, there is evidence that this process also occurs during psoriatic dermatitis and *S. pneumonia* infection of nasal mucosa (6, 17). Thus, local γδT17 cells may initiate inflammation, stimulating proliferation of LN γδT17 cells which then home to the target site in a second wave of innate-like IL-17 production. This working model (Figure 2) should be tested in additional pathophysiological settings, as again it is reminiscent of the human system where expansion of circulating γδT17 cells is documented during inflammation.

## Can we Translate Our Knowledge of γδT17 Cells from Mice to Humans?

**Figure 3:**
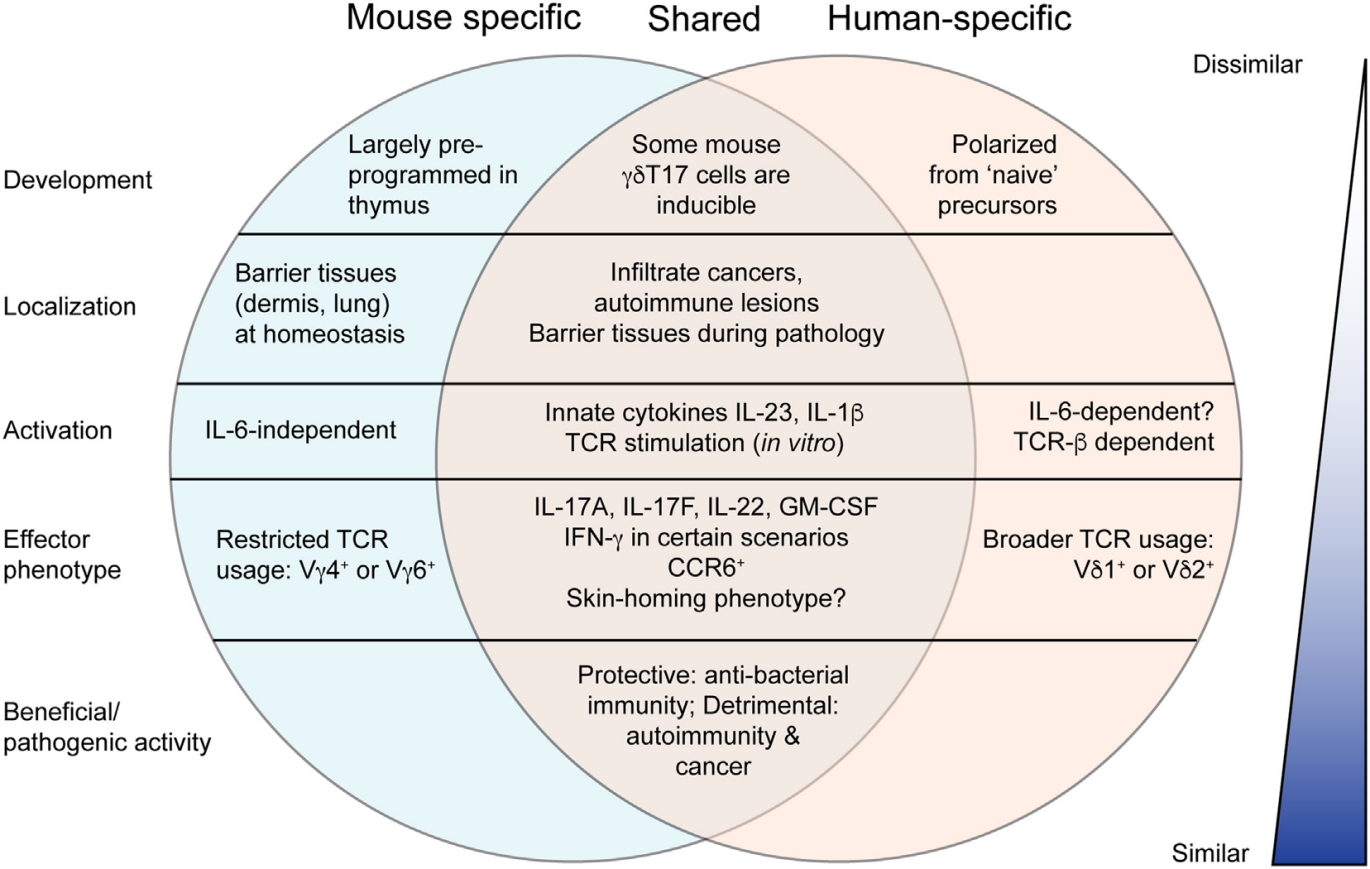
Comparison of murine and human γδT17 cells. Murine γδT17 cells are preprogrammed, tissue-localized innate-like sentinels that respond to innate cytokines. Human γδT17 cells are very rare and develop from naïve precursors upon T cell receptor (TCR) stimulation. However, there are many similarities between mouse and human as outlined. Most notably, mouse and human γδT17 cells are beneficial or pathogenic in highly similar scenarios, suggesting that knowledge from mice may be useful to promote antibacterial immunity or treat autoimmunity and cancer in humans. Moreover, further research to clarify emerging concepts discussed in this review may reveal greater similarity.

The relevance of extensive research into murine tissue-resident γδT17 cells may be questioned by their conspicuous absence in many human tissues. Moreover, key features of γδT17 cell biology in mice appear to clash with their rare human counterparts. While murine γδT17 cells gain their effector function in the thymus and can be subsequently activated independently of the TCR (2), human thymic γδ T cells are immature and γδT17 cells are presumably polarized from “naïve” peripheral blood precursors when provided with antigen, costimulatory, and inflammatory signals (49, 50, 86). These induced γδT17 cells express CCR6, RORγt, and receptors for IL-23 and IL-1β like their murine counterparts, as well as CD161, an NK receptor shared with human Th17 cells (50, 87). Human γδT17 cells do not appear to show the highly restricted TCR expression found in mice, as both those expressing typical peripheral blood Vγ9Vδ2 TCRs and tissue-biased Vδ1 TCRs (with varied and undefined γ chain pairing) have been identified in patient samples (50, 88). Accumulating evidence suggests that human γδT17 cells may perform similar functions to those in mice, including host defense and exacerbation of autoimmunity and cancer. In addition, some of the emerging concepts discussed earlier suggest that γδT17 cells may not be as different between species as initially thought (Figure 3).

Whereas mouse γδT17 cells comprise the majority of γδ T cells found in certain tissues (such as dermis, peritoneal cavity, and lung), γδ T cells in humans are largely IFN-γ producing and/or cytotoxic in function (7). However, γδT17 cells have been observed in some pathological scenarios that echo the mouse system. Human γδT17 cells have been identified in the cerebrospinal fluid of multiple sclerosis patients, and infiltrating colorectal and gallbladder cancer, similar to mouse γδT17 cells in EAE and cancer models (88–90). Human γδT17 cells have also been identified in lesional psoriasis skin, although not in healthy tissue (91). Circulating γδT17 cells are rare in healthy individuals but are present in bacterial meningitis patients and disappear upon successful treatment (50). Moreover, they are elevated in the peripheral blood of patients with active tuberculosis or HIV (92, 93). Thus, while γδT17 cells are particularly rare in healthy humans, it is likely that they will prove relevant in wider infectious and pathological settings upon further investigation.

It is also possible that the equivalent population of mouse γδT17 cells in humans is not necessarily defined by IL-17 production. After all, many alternative traits identify mouse γδT17 cells, such as expression of specific homing molecules, activation markers and other subset-specific surface markers, participation in particular immune responses and specific tissue localization. It is important to consider that immune cell populations are generally named in reference to an effector molecule or function relevant at the time of their discovery, and not necessarily that most critical to their function which may become evident in light of further research. For example, Th17 cells were named after their production of IL-17, although in the context of autoimmunity, this nomenclature may be misleading as it is their production of GM-CSF that may contribute more to their pathological function (94, 95). With this in mind, consider that IL-17-producing γδ T cells are scarce in healthy human skin or blood. However, a significant proportion of circulating γδ T cells in healthy individuals expresses skin-homing molecules such as CLA, CCR4, CCR6, and CCR10 (96). Moreover, it is these cells that home to psoriatic skin, and in doing so decrease in blood frequency. Therefore, are these CCR6^+^ γδ T cells equivalent to mouse γδT17 cells? Further investigation is clearly warranted, perhaps first by performing comparative transcriptomic analyses.

A non-mutually exclusive alternate explanation for the discrepancy between mouse and human γδT17 cells is that Type 3 innate lymphoid cells (ILC3s) in humans have evolved to fill the niche occupied by γδT17 cells in mice. Already in the mouse there is substantial overlap in the functions of ILC3s and γδT17 cells: both are tissue-localized, innate-like responders to bacterial infection with preprogrammed IL-17- and IL-22-secreting effector function (97, 98). Apart from a slight differential bias in specific tissue responses, such as the preferential involvement of ILC3s in intestinal protection or γδT17 cells in skin infection, it is possible that the only basic features distinguishing the functional niche of these populations are the emerging concepts discussed throughout this review. Without a thorough understanding of γδT17 cell TCR responses, memory, migratory behavior, and functional plasticity, it is unclear why γδT17 cells and ILC3s have co-evolved in the mouse. In humans, where the TCR plays a more obvious role in γδT17 cell biology, it is conceivable that ILC3s have evolved to occupy the entire innate(-like) IL-17 effector niche. Perhaps it is most pertinent to further investigate inducible murine γδT17 cells, as these appear to more closely reflect their human counterparts. Given the dearth of information about human γδT17 cells, whether the emerging themes of mouse γδT17 cell biology outlined here could be exploited for therapeutic benefit will require a more focused effort to extend findings in mice to humans (Box 1).

**Box 1. Research priorities in γδT17 cell biology.**

1. Defining, if any, the *in vivo* antigens recognized by mouse/human γδT17 cells
2. Determining the relative influence of T cell receptor vs. inflammatory cytokine signaling in γδT17 (patho)physiological responses, in mouse and human
3. Establishing whether γδT17 cells are resident in normal human tissues. If so, do they develop from naïve precursors upon inflammation or are they preprogrammed?
4. Clarifying the extent of interplay between tissue-localized and circulating γδT17 cells
5. Assessing whether γδT17 cells are capable of mounting *bona fide* memory responses to pathogens
6. Investigating the extent of γδT17 cell plasticity and how it influences immunity

## Concluding Remarks

The γδT17 cell subset, discovered just over 10 years ago, is proving more and more complex and intriguing every year. This review has given an overview of the key emerging concepts that may improve our understanding of how γδT17 cells fit into the grand scheme of tissue immunity. Thus, by considering the latest trends in immune–microbiota interactions, immunometabolism, and single cell transcriptomics, we may soon clarify where γδT17 cells sit on the innate/adaptive spectrum. This will likely explain why they constitute a major source of IL-17 at particular stages of multiple experiment models of disease, and their non-redundant roles in relation to ILC3s and Th17 cells. Finally, we strongly believe that our improved knowledge of murine γδT17 cells will carry across to their human counterparts, and thus be exploited for clinical benefit.

## Author Contributions

DM conceptualized the review, wrote the manuscript, and prepared figures. BS-S, IC, and SM conceptualized the review, provided essential discussion, and edited the manuscript.

## Conflict of Interest Statement

The authors declare that the research was conducted in the absence of any commercial or financial relationships that could be construed as a potential conflict of interest.
